# Over-expression of SRD5A3 and its prognostic significance in breast cancer

**DOI:** 10.1186/s12957-021-02377-1

**Published:** 2021-08-31

**Authors:** Yong-ping Zhang, Wen-ting Na, Xiao-qiang Dai, Ruo-fei Li, Jian-xiong Wang, Ting Gao, Wei-bo Zhang, Cheng Xiang

**Affiliations:** 1 Department of Vascular and Thyroid Surgery, Xianyang Central Hospital, Xianyang, 712000 Shanxi China; 2 Department of Anesthesiology, Xi’an Yanliang Railway Hospital, Xi’an, 710089 Shanxi China; 3 Department of Orthopaedics, Xianyang Central Hospital, Xianyang, 712000 Shanxi China; 4 Department of Pulmonary and Critical Care Medicine, Xianyang Central Hospital, Xianyang, 712000 Shanxi China; 5 Department of Pathology, Xianyang Central Hospital, Xianyang, 712000 Shanxi China; 6 Department of Thyroid Surgery, The Second Affiliated Hospital of Zhejiang University School of Medicine, 88 Jiefang Road, Hangzhou, 310009 Zhejiang China

**Keywords:** SRD5A3 expression, Breast cancer, Prognosis, Data mining

## Abstract

**Objective:**

The study aimed to compare the Steroid 5 alpha-reductase 3 (SRD5A3) expression levels in breast cancer (BC) and normal tissues, to investigate the prognostic value of SRD5A3 mRNA expression in BC patients and to identify the SRD5A3-related signaling pathways using bioinformatics approaches.

**Methods:**

We evaluated the expression levels of SRD5A3 and survival data in BC patients using different bioinformatic databases. Further, Cox regression analysis was conducted to predict the independent prognostic factors for BC. Moreover, the association of SRD5A3 with clinicopathological factors was measured through LinkedOmics database. And the potential role of SRD5A3 was determined by Gene Ontology and KEGG pathway enrichment analysis. Finally, protein network of SRD5A3 was constructed and genetic alterations were analyzed.

**Results:**

Bioinformatic data indicated that both mRNA and protein expression levels of SRD5A3 were higher in BC group than those in the normal group (*P* < 0.05). Besides, BC patients with higher SRD5A3 mRNA expression levels had a lower overall survival (all *P* < 0.05). Cox regression analysis further demonstrated the independent prognostic value of SRD5A3 in BC (*P* = 0.015). SRD5A3 mRNA expression was significantly associated with N stage (*P* < 0.001), age (*P* < 0.05), and histologic subtype (*P* < 0.001) but had no significant relationship with other clinical characteristics (all *P* > 0.05). Moreover, the functional enrichment analysis revealed that the SRD5A3 was involved in metabolism-related pathways (all *P* < 0.05).

**Conclusions:**

SRD5A3 was highly expressed in BC tissues and high SRD5A3 expression was related to poorer prognosis. SRD5A3 serves as an oncogene and might function as a potential biomarker for prognosis and a therapeutic target for BC.

## Introduction

Breast cancer (BC) is one of the most common malignancies among women, accounting for 20% of all cancers and 22% of deaths [1]. It is classified into three subtypes: triple-negative breast cancer (TNBC), human epidermal growth factor receptor 2-positive (HER2+) BC, and estrogen receptor-positive (ER+)/luminal BC [2]. In the USA, one in eight women is diagnosed with BC, resulting in more than 252,710 new cases of this disease each year [3]. In China, BC is the most prevalent cancer among females, and the number of new cases diagnosed is increasing every year [3]. The improvement of the BC treatment has been achieved in the last decade, including radiation, chemotherapy and endocrine therapies, and targeted therapies. However, some patients have failed or recurred in targeted therapies, promoting the ongoing search for novel prognostic markers [4]. Therefore, it is of great significance to develop potent biomarkers to improve the clinical prognosis of BC patients.

As a protein coding gene, Steroid 5 alpha-reductase 3 (SRD5A3) is a member of the SRD5A family, which plays a regulatory role in male sexual development and the production of steroid hormones by catalyzing the conversion from testosterone into the most potent natural androgen 5 alpha-dihydrotestosterone [5]. It is an important molecule in glycosylation metabolism and steroid hormone formation [6]. It has been reported that SRD5A3 had a higher expression in prostate cancer, endometrial cancer and human fetal liver [7–9]. Recent research has demonstrated that high SRD5A3 expression facilitated tumor growth and led to poor survival in human hepatocellular cancer (HCC) [6]. Nevertheless, few reports described the expression of SRD5A3 in BC and little is known to the role of SRD5A3 in BC.

In this study, the Human Protein Atlas (HPA) database and Oncomine and Gepia databases were used to obtain the SRD5A3 expression levels. Further, the prognostic value of SRD5A3 mRNA expression in BC was evaluated by Gepia, which was validated by Kaplan-Meier plotter analysis. Then, the association of SRD5A3 mRNA expression with clinicopathological factors was analyzed through LinkedOmics database. We obtained the co-expression genes of SRD5A3 in cBioPortal database and conducted functional enrichment analysis in the David database. Finally, the protein network of predicted associations for SRD5A3 and alterations were investigated.

## Material and methods

### The localization of SRD5A3 protein expression in human tumor cells

HPA database (https://www.proteinatlas.org/) was used to derive the general protein expression profile of SRD5A3 in human tumor cells. The aim of HPA database is to map all the human proteins in cells, tissues, and organs using an integration of various omics technologies. The HPA database provides information on the tissue and cell distribution of 24,000 human proteins. Immunohistochemical techniques are used to detect the expression and distribution of each protein in normal and tumor tissues. We searched “SRD5A3” in HPA database and chose the tab “Cell” to acquire the localization of SRD5A3 protein in human tumor cells.

### SRD5A3 mRNA expression in various tumors

Oncomine database (https://www.oncomine.org/resource/main.html) was employed to acquire the mRNA expression levels of SRD5A3 in different cancers. Currently, Oncomine database is the largest oncology gene chip database and integrated data platform. SRD5A3 mRNA expression levels in tumor and normal tissues from different datasets were shown by setting *P* value < 0.05, fold change > 2, and gene rank top 10% as the parameters. Following this, we selected the tumor type “Breast cancer” and the SRD5A3 mRNA expression in three sub-studies was obtained for the subsequent meta-analysis.

### SRD5A3 expression levels in BC and normal tissues

We adopted Gepia database (http://gepia.cancer-pku.cn/detail.php) for analyzing SRD5A3 mRNA expression in BC tissues and normal tissues. GEPIA is an online tool for the analysis of the RNA sequencing expression data of 9736 tumors and 8587 normal samples from the TCGA and the GTEx projects, using a standard processing pipeline. Firstly, we entered into the Gepia database and chose “General”, followed by inputting the differential gene SRD5A3, and then the SRD5A3 gene expression profile across all tumor samples and paired normal tissues would be exhibited. Further, the SRD5A3 protein expression was studied through HPA database. We searched “SRD5A3” and chose “Pathology” to obtain representative immunohistochemical images and detailed information about SRD5A3 in BC and normal tissues.

### Relationship between SRD5A3 mRNA expression and clinical prognosis

First, the association of SRD5A3 mRNA expression with BC patient OS was assessed by Gepia database. OS is defined as the time from targeted agent administration to date of death or last contact [10]. We chose “Survival” and setting the expression median as the group cutoff. After that, Kaplan-Meier plotter database (http://kmplot.com/analysis) was used to evaluate the correlation between SRD5A3 gene expression and survival time. The Kaplan-Meier plotter database is an online survival analysis platform that evaluates the effect of mRNA expression levels of genes of interest on prognosis in patients with specific tumors. We chose “Breast cancer” in “mRNA gene chip” and searched the “SRD5A3” gene. In order to keep the consistency with the group cutoff value in Gepia, we chose the “median” to divide the groups. The survival curves of patients with high and low SRD5A3 expression in BC were drawn respectively. Further, clinical information and expression data of BC were retrieved from cBioPortal database (https://www.cbioportal.org/) by searching “BRCA” and choosing “Breast Invasive Carcinoma” (TCGA, Firehose Legacy) with 1108 samples for Cox regression analysis. Patients with complete survival and expression data were enrolled in the study. Male is the reference level for gender, stage 1 for stage, and luminal for histologic subtype.

### Relationship between SRD5A3 gene expression and clinicopathological factors

LinkedOmics database (http://linkedomics.org/login.php) was used for evaluation of SRD5A3 expression in BC patients and clinicopathological characteristics. LinkedOmics is a publicly available portal that includes multi-omics data from all 32 TCGA Cancer types, which can be used for assessing the correlation of target gene and clinicopathological factors.

### Gene Oncology (GO) annotation and Kyoto Encyclopedia of Genes and Genomes (KEGG) pathway analyses

SRD5A3 co-expressed genes were identified using cBioPortal database. We searched “BRCA” and chose the “Breast Invasive Carcinoma (TCGA, Firehose Legacy)” and clicked “Query by Gene”; then, we entered “SRD5A3” gene and chose “Co-expression”. Totally 200187 co-expressed genes were shown and finally 69 genes with high correlation were screened out for functional enrichment analysis with *q*-value ≤ 0.05, absolute value of Spearman’s *R* > 0.3 as a threshold. Next, the selected co-expressed genes were loaded into the David website (https://david.ncifcrf.gov/tools.jsp) for GO analysis and KEGG pathway analysis.

### Construction of protein-protein interaction (PPI) network

STRING (https://string-db.org/) is a database of known and predicted protein-protein interactions, including physical and indirect functional associations from computational prediction, knowledge transfer between organisms, and other databases. The database was used to explore the structural proteins associated with SRD5A3 function by inputting “SRD5A3” and setting “Homo sapiens” as criterion.

### Genetic alteration analysis

We have demonstrated that there was a relation between SRD5A3 and BC via Gepia and Kalan-Meier plotter analyses. To provide a theoretical basis for further research, SRD5A3, DOLK, SRD5A1, and HSD17B3 genomic changes in BC were investigated by cBioPortal online tool (TCGA, Firehose Legacy, 1108 samples). The selected genomic profiles were “Mutations”, “Putative copy-number alterations from GISTIC”, and “mRNA expression z-scores relative to diploid samples (RNA Seq V2 RSEM)”. Gene set is as follows: SRD5A3, DOLK, SRD5A1, and HSD17B3. The alteration frequency was summarized in “Cancer Types Summary”. The specific alteration of each gene and SRD5A3 mutation were exhibited in “OncoPrint” and “Mutations”, respectively.

### Statistical analysis

SPSS23.0 (SPSS, Inc., Chicago, IL, USA) software was used for all statistical analyses. Image J software was employed to quantify the amount of SRD5A3 protein in carcinoma and normal tissues. The relationship between SRD5A3 expression and clinicopathological parameters from LinkedOmics database was evaluated by Kruskal-Wallis test, Wilcox test, and Spearman correlation. Kaplan-Meier survival analyses were performed using the log-rank test to explore the correlation between SRD5A3 mRNA expression and OS in BC. *P* < 0.05 was considered to be statistically significant.

## Results

### The general protein expression profile of SRD5A3 in human tumor cells

**Fig. 1:**
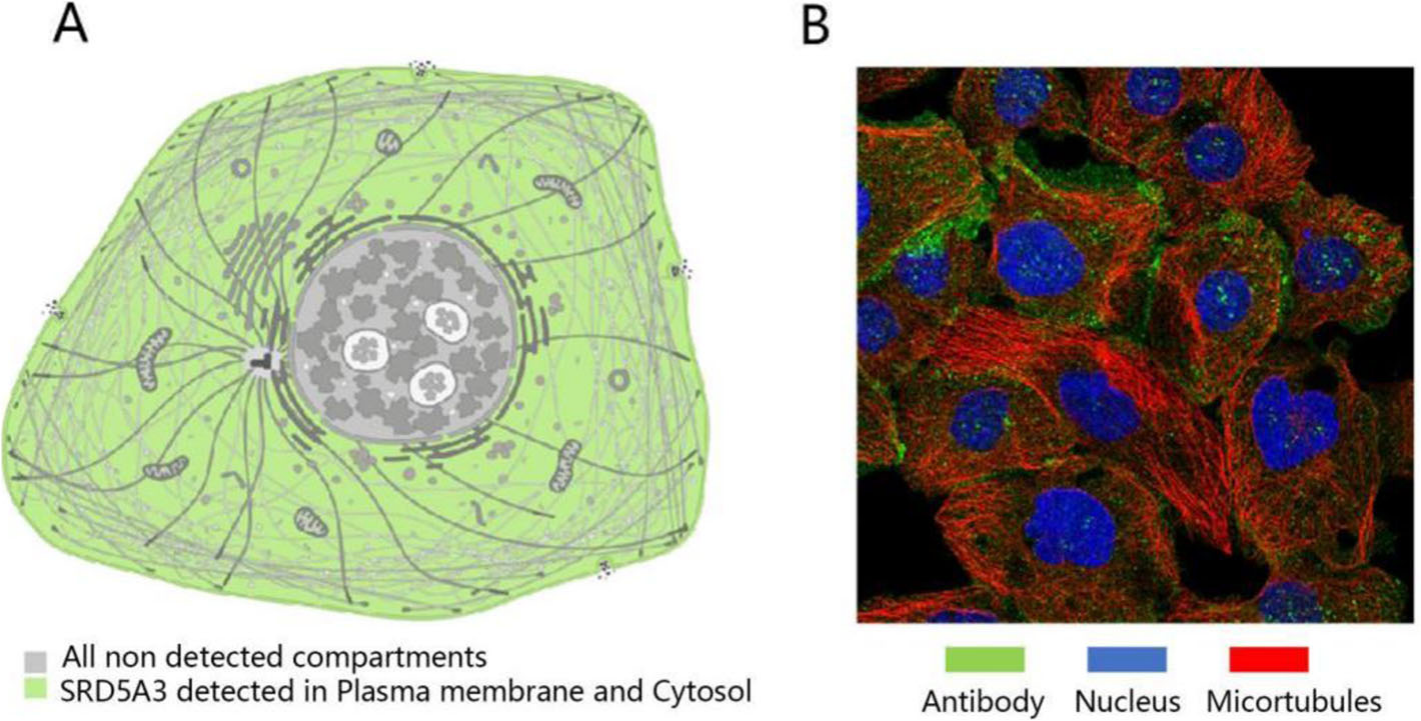
The general protein expression profile of SRD5A3 in human tumor cells. **A** Localization of SRD5A3 protein expression in tumor cells. **B** Immunofluorescence staining of SRD5A3 in tumor cells

To visualize the localization of SRD5A3 protein in human tumor cells, we retrieved “SRD5A3” in the “Cell” retrieval tab in the HPA database. The results showed that SRD5A3 protein is expressed in the plasma membrane and cytosol, while no SRD5A3 expression is detected in the nucleus (Fig. 1A). The SRD5A3 immunofluorescence staining result in tumor cells was shown in Fig. 1B.

### SRD5A3 over-expression in BC

**Fig. 2:**
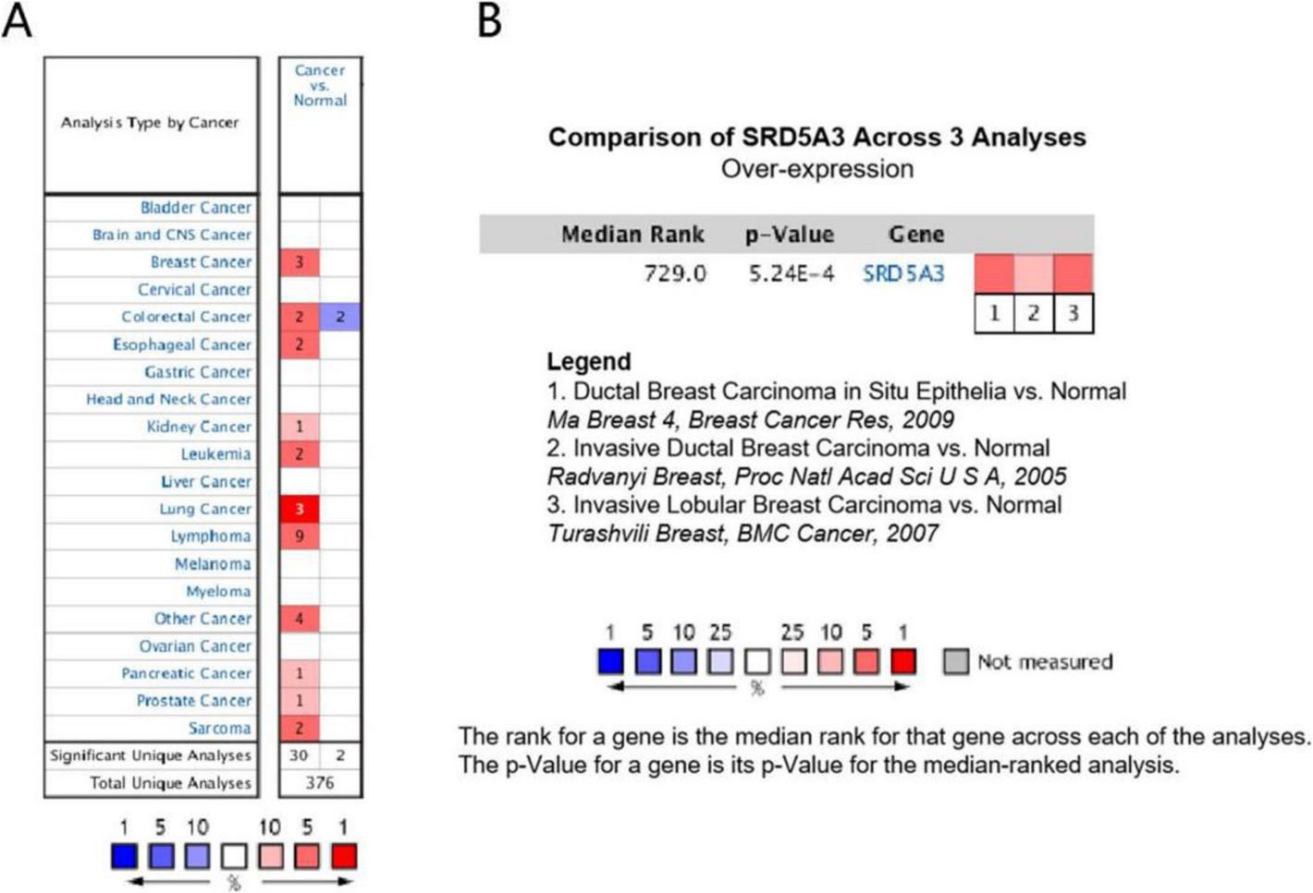
SRD5A3 mRNA expression in different cancers. **A** Database of SRD5A3 mRNA expression in human tumors. Compared with the normal group, “blue” represents low expression in tumor group, and “red” means high expression in tumor group. Number, the number of studies. The parameters were *P* value < 0.05, fold change > 2, and gene rank top 10%. **B** Comparison of SRD5A3 across 3 analyses by meta-analysis. All data were obtained from Oncomine database

To investigate the expression of SRD5A3 in BC, we first visualized the mRNA expression of SRD5A3 in human cancer and normal specimens using the Oncomine database. Compared with the normal tissues, SRD5A3 mRNA was highly expressed in most of the cancers including BC. Only two studies reported that low SRD5A3 expression was observed in colorectal cancer (Fig. 2A). Then, the gene expression of SRD5A3 in BC was investigated. Three studies all showed high expression of SRD5A3 in BC, and a meta-analysis of these three studies was conducted. The results showed that the expression of SRD5A3 was increased significantly in BC tissues, and the difference was statistically significant (Fig. 2B). The research information of the three datasets, including *P* value, fold change, and sample size, is shown in Table 1.

**Table 1:** SRD5A3 expression in breast cancer

Cancer type	*P*-value	Fold change	Rank (10%)	Sample	Reference
Invasive lobular breast carcinoma	0.016	3.474	10	15	Turashvili et al. [11]
Ductal breast carcinoma in situ	< 0.001	2.924	10	48	Ma et al. [12]
Invasive ductal breast carcinoma	0.016	4.788	10	63	Radvanyi et al. [13]

**Fig. 3:**
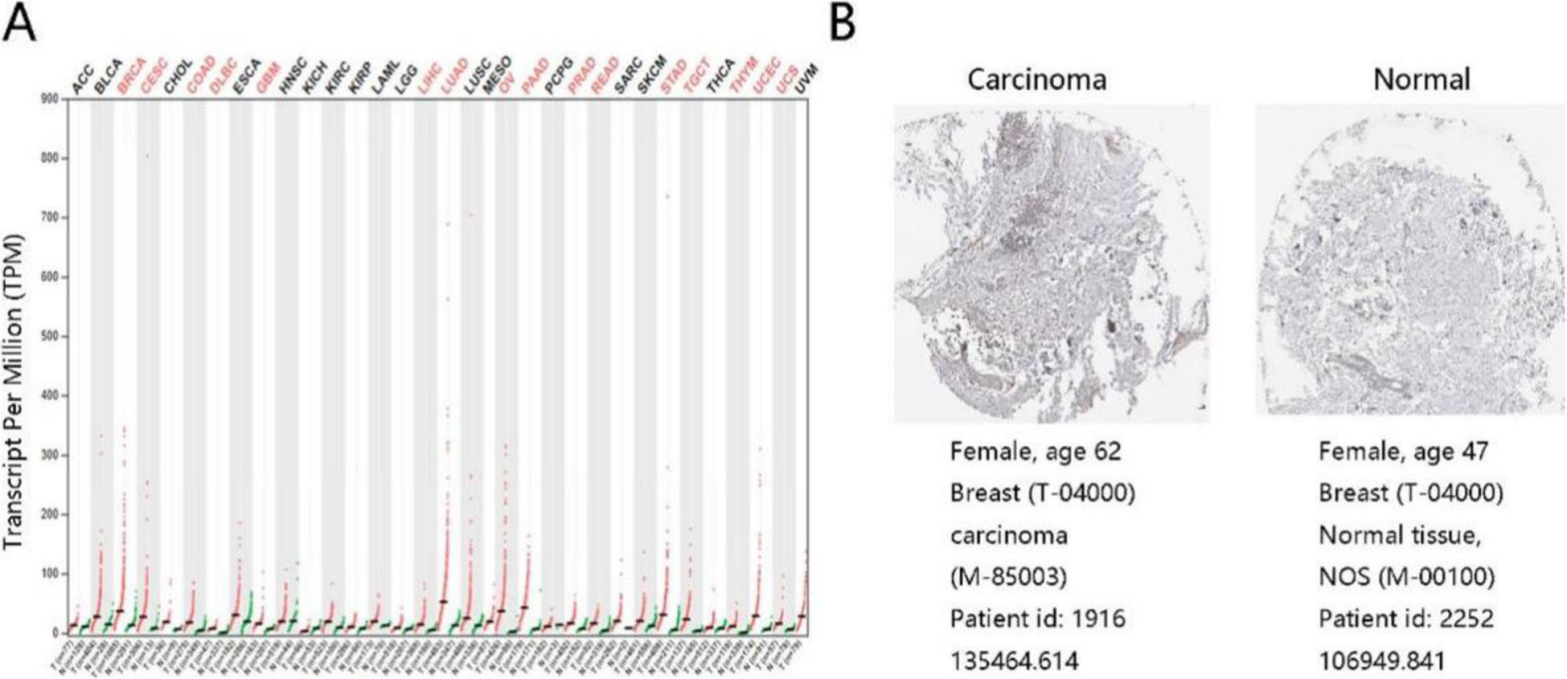
SRD5A3 expression in breast cancer and normal tissues. **A** The SRD5A3 mRNA expression profile across all tumor samples and paired normal tissues. Compared with the normal group, “red” represents high expression in cancer group with significant difference, and “black” stands for no statistical significance between two groups. Data were derived from the Gepia database. **B** Representative immunohistochemistry images and relative information on SRD5A3 protein in breast cancer and normal tissues from the HPA database. The amount of SRD5A3 protein was quantified through Image J software

The differential expression of SRD5A3 in clinical tumor tissues and normal tissues was further verified in Gepia database and HPA database. The results of Gepia database showed that the mRNA expression of SRD5A3 in BC tissues was significantly higher than that in normal tissues, and the difference was statistically significant (Fig. 3A). Similarly, HPA database results suggested that SRD5A3 protein expression was highly expressed in BC tissues (Fig. 3B). The detailed cell information was shown in Table 2.

**Table 2:** Detailed cell information in breast cancer and normal breast

Group	Staining	Intensity	Quantity	Location
Carcinoma	Low	Moderate	< 25%	Cytoplasmic/membranous
Normal	Not detected	Negative	None	None

### High mRNA expression of SRD5A3 gene predicts poor prognosis

**Fig. 4:**
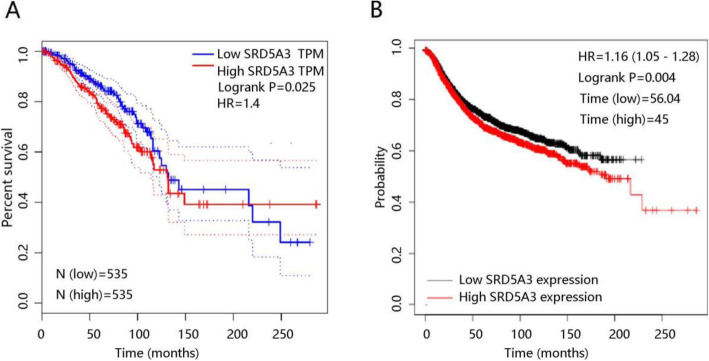
Association of SRD5A3 mRNA expression with clinical prognosis. **A** Gepia database. **B** Kaplan-Meier plotter analysis

In order to evaluate the effect of SRD5A3 expression on the prognosis of patients, the analysis was first conducted in Gepia database, and the results indicated that patients with high SRD5A3 mRNA expression had a poorer clinical prognosis with significant difference (*P* = 0.025) (Fig. 4A). Kaplan-Meier plotter analysis and log-rank test were conducted to further verify the correlation between SRD5A3 mRNA expression and OS. The survival analysis results showed that high SRD5A3 expression cohort had a poorer prognosis and a shorter survival time of 45 months in comparison with low SRD5A3 expression cohort who had survival time of 56.04 months (*P* = 0.004) (Fig. 4B). These findings revealed that SRD5A3 may serve as a potential new indicator of prognosis in BC patients.

We further investigated the risk factors influencing the clinical outcome of BC. Since no death occurred in stage 1 and stage 2, univariate analysis of stage 2 was not performed. Univariate analysis results showed that age, stage 3, triple-negative subtype, and SRD5A3 were significantly related to OS in BC patients (all *P* < 0.05), while gender, stage 4, and HER2+ subtype had no remarkable relationship with OS in BC. When integrating these factors into multivariate analysis, age (HR = 1.039, *P* < 0.001), and SRD5A3 (HR = 1.865, *P* < 0.05) were still independent prognostic parameters for BC (Table 3). The above results indicated that SRD5A3 can be considered a promising independent prognostic biomarker in BC.

**Table 3:** Cox regression analysis of SRD5A3 and other clinicopathological factors in BC patients

Covariates	Univariate analysis			Multivariate analysis			
	HR	95% CI	*P*-value	HR	95% CI		*P*-value
Age	1.018	1.005–1.030	0.004	1.039	1.020–1.059		< 0.001
Gender female	0.429	0.136–1.349	0.148	1.137	0.258–5.010		0.866
Stage 2	NA	NA	NA	1.268	0.000–4.583E+ 43		0.996
Stage 3	68.847	17.534–270.327	< 0.001	211,065.668	0.000–1.280E+ 43		0.782
Stage 4	823,685.985	0.000–2.511E+ 72	0.862	184,003.037	0.000–1.125E+ 43		0.785
HER2 +	0.468	0.115–1.907	0.289	0.264	0.063	1.101	0.068
Triple-negative	0.327	0.132–0.810	0.016	0.545	0.217	1.366	0.195
SRD5A3	1.000	1.000–1.000	0.014	1.865	1.129	3.081	0.015

*95% CI* 95% confidential interval

### Association of SRD5A3 mRNA expression with clinicopathological characteristics in BC patients

**Fig. 5:**
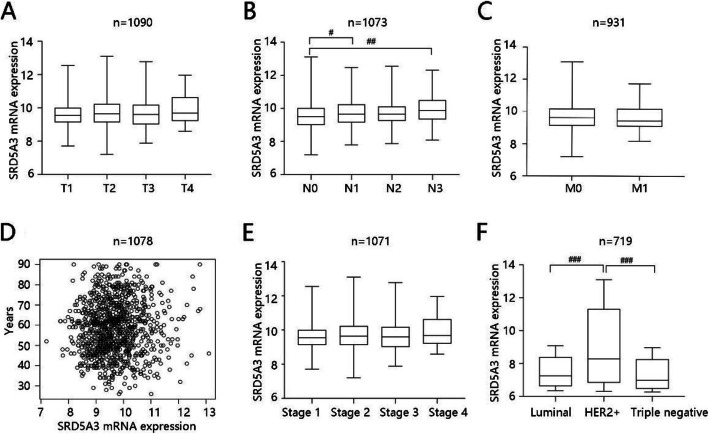
Association of SRD5A3 mRNA expression with clinicopathological characteristics. **A** T stage. **B** N stage. **C** M stage. **D** Age. **E** Pathological stage. **F** Histologic subtype. ^#^*P* < 0.05, ^##^*P* < 0.01, ^###^*P* < 0.001

We have found that high SRD5A3 mRNA expression predicted poor prognosis, and hence we explored the clinicopathological factors that could affect SRD5A3 mRNA expression via LinkedOmics database. The results showed that SRD5A3 gene expression was closely related to N stage (*P* < 0.001), age (*P* < 0.05), and histologic subtype (*P* < 0.001) but had no significant difference with T stage, M stage, and pathological stage (all *P* > 0.05) (Fig. 5A–F). Notably, patients in N0 stage were significantly different with those in N1 and N3 stage at SRD5A3 mRNA expression level (*P* < 0.05) (Fig. 5B). Besides, patients with HER2+ BC had highest SRD5A3 mRNA expression, which had statistical difference compared to those with luminal BC and TNBC (*P* < 0.001) (Fig. 5F). Detailed information including statistical methods, sample size, and statistical values were shown in Table 4.

**Table 4:** Relationship of SRD5A3 mRNA expression and clinicopathological factors in breast cancer

Clinicopathological factors	Statistical method	Sample	Statistic	*P*-value
T stage	Kruskal-Wallis test	1090	3.614	0.3063
N stage	Kruskal-Wallis test	1073	19.32	< 0.001
M stage	Wilcox test	931	− 0.02687	0.4695
Age	Spearman correlation	1078	0.06488	0.03672
Pathologic stage	Kruskal-Wallis test	1071	6.998	0.07197
Histologic subtype	Kruskal-Wallis test	719	26.82	< 0.001

### GO and KEGG pathway analyses of SRD5A3

To investigate the potential role of SRD5A31 in BC, we obtained 200,187 SRD5A3 co-expressed genes from the cBioPortal database. According to *q*-value ≤ 0.05 and absolute value of Spearman’s *R* > 0.3, 69 co-expressed genes were screened out for subsequent GO and KEGG pathway analyses. The top genes with the highest correlation were TMEM165, SAR1B, and GGCT. The results of GO analysis showed that these co-expressed genes were mainly enriched in the following: oxidation-reduction process, regulation of cellular amino acid metabolic process, glutathione metabolic process, ER to Golgi vesicle-mediated transport, COPII vesicle coating, NIK/NF-kappaβ signaling, endoplasmic reticulum, endoplasmic reticulum, mitochondrial inner membrane, and Golgi membrane which is in consistent with the previous results that the SRD5A3 protein expression is located in cytoplasm and cell membrane in human tumor cells (Table 5).

**Table 5:** GO annotation and KEGG pathway analyses of genes co-expressed with SRD5A3 in breast cancer

Category	ID	Term	Count	*P*-value
GO annotation	0055114	Oxidation-reduction process	8	0.00548
	0006521	Regulation of cellular amino acid metabolic process	3	0.0148
	0006749	Glutathione metabolic process	3	0.0176
	0006888	ER to Golgi vesicle-mediated transport	4	0.0204
	0048208	COPII vesicle coating	3	0.0207
	0038061	NIK/NF-kappaβ signaling	3	0.0240
	0005783	Endoplasmic reticulum	11	< 0.001
	0005789	Endoplasmic reticulum membrane	11	< 0.001
	0005743	Mitochondrial inner membrane	8	< 0.0014
	0000139	Golgi membrane	8	0.00457
KEGG pathway	00480	Glutathione metabolism	4	< 0.001
	03050	Proteasome	3	0.0127
	01130	Biosynthesis of antibiotics	4	0.0490
	01100	Metabolic pathways	9	0.0903

In order to study the signaling pathways in which SRD5A3 and its co-expressed genes may be involved, we analyzed the KEGG pathway of these co-expressed genes on the website of David. Analysis results showed that these genes were mainly involved in 3 signaling pathways, including glutathione metabolism, proteasome, and biosynthesis of antibiotics. Among them, glutathione metabolism pathway was the most significant pathway (*P* < 0.001) (Table 5).

### SRD5A3 predicted PPI analysis

**Fig. 6:**
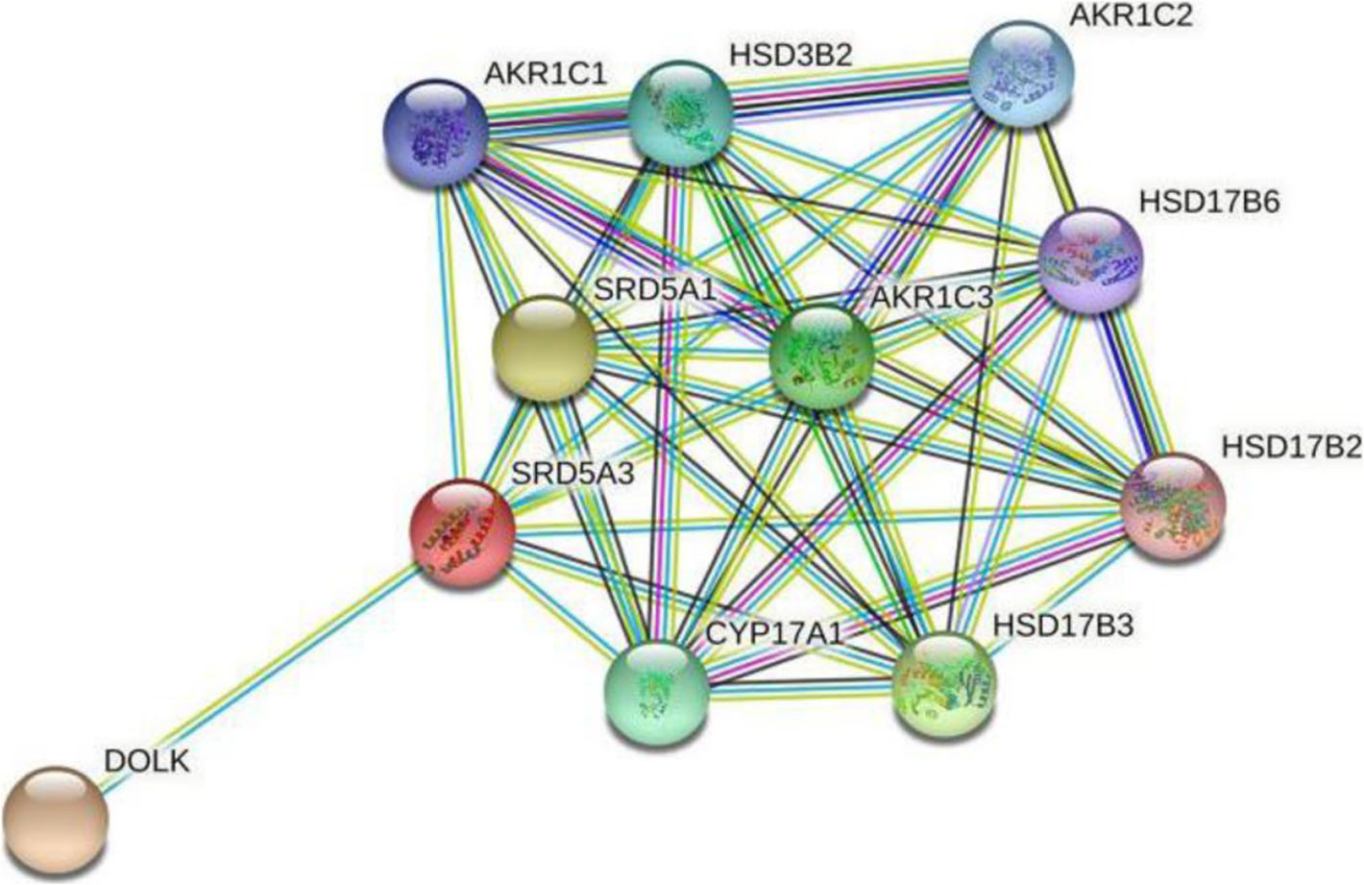
Structural proteins related to SRD5A3 function. Network nodes represent proteins; edges represent protein-protein associations

In order to further understand the value of SRD5A3 in BC, PPI network was constructed through STRING database. The results showed that ten predicted functional partners were DOLK, SRD5A1, HSD17B3, AKR1C3, CYP17A1, HSD3B2, AKR1C2, AKR1C1, HSD17B6, and HSD17B2, whose correlation score generated from STRING database were 0.987, 0.979, 0.956, 0.945, 0.942, 0.940, 0.936, 0.936, 0.936, 0.932, and 0.930, respectively (Fig. 6). DOLK is involved in the protein binding, transferase activity, and metabolism of proteins. SRD5A1 is participated in oxidoreductase activity and metabolism pathway. And HSD17B3 is mainly enriched in oxidoreductase activity as well as metabolism pathway. These three proteins were found to have highest correlation with SRD5A3, and hence need more in-depth analysis.

### Genetic alteration analysis

**Fig. 7:**
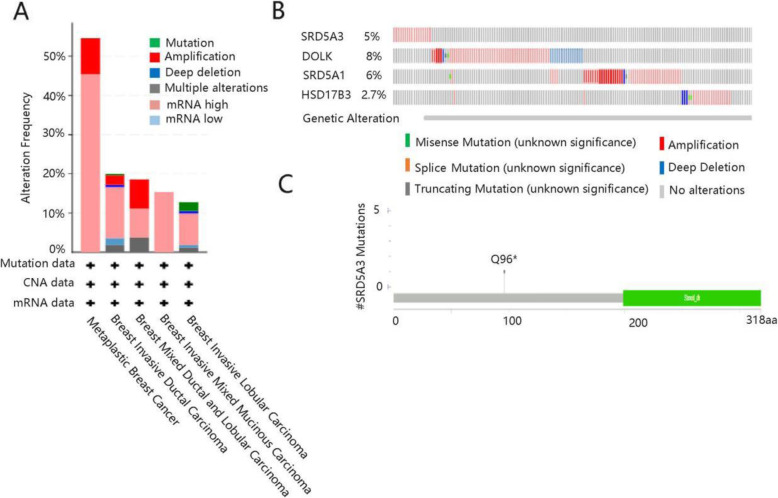
Genetic alteration and mutation analyses. **A** The alteration frequency of SRD5A3, DOLK, SRD5A1, and HSD17B3 in breast cancer. **B** Specific alteration percentage of SRD5A3, DOLK, SRD5A1, and HSD17B3 in breast cancer. **C** SRD5A3 mutation site in breast cancer

Since irreparable structural mutations in cells may result in cancer occurrence, we then determined the genetic alterations of SRD5A3, DOLK, SRD5A1, and HSD17B3 in BC. The alteration frequency in BC subtypes was summarized in Fig. 7A. As shown in Fig. 7B, the alteration percentage of these four genes varied from 2.7 to 8% (SRD5A3, 5%; DOLK, 8%; SRD5A1, 6%; HSD17B3, 2.75), and the alterations included missense mutation, splice mutation, truncating mutation, amplification, and deep deletion. In “Mutations”, the result showed that SRD5A3 mutation site was located at Q96* with the characteristics of nonsense mutation and diploid copy type (Fig. 7C). The authors speculated that the mutation of SRD5A3 may upregulate the SRD5A3 expression, and hence leading to BC.

## Discussion

Our study revealed that SRD5A3 protein is expressed in plasma membrane and cytosol. Both mRNA and protein expression levels of SRD5A3 were highly expressed in the BC group compared with the normal group. Besides, high SRD5A3 mRNA expression in BC patients predicts poorer prognosis with lower OS time. We also found that SRD5A3 mRNA expression was related to N stage, age, and histologic subtype but had no remarkable relationship with T stage, M stage, and pathological stage. KEGG analysis showed that the genes co-expressed with SRD5A3 were involved in the metabolic pathways.

Breast cancer belongs to one of the most common malignancies, which is the second leading cause of death from cancer in females. Presently, several important biomarkers have been proved to participate in BRCA progress. Yong et al. proved that miR-381-3p inhibited BC progression and epithelial-mesenchymal transition [14]. Yu et al. demonstrated that miR-92b-3p expression was increased in BC patients and was closely related to the clinical staging and degree of differentiation in BC [15]. In addition, the importance of some chimeric genes [16] and methylation regulated gene such as m^5^C [17] in cancer development and progression has been reported as well. Interestingly, intra-mammary lymph nodes may serve as a BC prognostic tool, which was overlooked in the clinical and radiological examinations [18]. While study also showed that axillary lymph node dissection could be avoided in a specific population of sentinel lymph node-positive patients [19]. More and more biomarkers and tools have been revealed in the field of BRCA prevention and treatment.

SRD5A3 is located on chromosome 4 (about 36 kDa in length) in the human genome [20]. Previous study has proved that over-expression of SRD5A3 led to the occurrence and development of HCC by in vitro and in vivo experiments and HCC patients with higher SRD5A3 expression had poorer OS [6]. And SRD5A3 was highly expressed in hormone-refractory prostate cancer tissues compared with the normal tissues [21]. Gene expression profile has been widely used to highlight the underlying transcriptional programs and molecular mechanisms between malignant and normal conditions [22]. Our study observed that SRD5A3 mRNA expression was over-expressed in many tumors; both the mRNA and protein expression levels of SRD5A3 were higher in BC by using bioinformatics approaches. And patients with higher SRD5A3 expression had lower OS, suggesting that high SRD5A3 mRNA expression contributes to poorer prognosis. Our results also showed that the patients with HER2+ BC had highest SRD5A3 mRNA expression among the three subtypes, which may be the cause of the worse OS in HR-/HER2+ patients [23]. It followed that the prognostic impacts of SRD5A3 may be related to receptor activity in BRCA. Previous study has revealed that CCNE1 over-expression confers a poorer prognosis in TNBC [24], which also supported the prognostic significance of receptor activity in BRCA. The effects of receptor activity on BRCA patient’s survival need further confirmation.

After that, we selected 69 genes co-expressed with SRD5A3 through cBioPortal database, and the co-expressed genes were closely related to SRD5A3 including TMEM165, SAR1B, and GGCT. Murali et al. showed that knockdown of TMEM165 suppressed the tumor growth of BC in vitro and the increased TMEM165 expression led to reduced OS [25]. It has also been proved that removal of SAR1B inhibited the proliferation and induced apoptosis of colorectal cancer cells [26]. In addition, loss of TMEM165 was revealed to inhibit migration and invasion of BC cells [25]. Therefore, SRD5A3 and its co-expressed genes were closely related to the occurrence and development of tumors and affect the prognosis of patients.

In addition, KEGG pathway analysis showed that SRD5A3 was associated with glutathione metabolism, proteasome, biosynthesis of antibiotics, and metabolic pathways. Glutathione represents a crucial role in various biological functions, such as nutrient metabolism and antioxidant defense, while deregulation of its synthesis may result in pathogenesis [27]. Further, the enhanced glutathione levels are exhibited in some cancers and have been reported to confer tumor resistance [28]. Moreover, previous metabolic studies have revealed that when nutrients, such as lipids, proteins, and nucleic acids, were abundant, oncogenic signaling pathways directly enhanced nutrient acquisition, and facilitated cancer cell proliferation [29, 30]. Thus, the authors speculate that high expression of SRD5A3 may promote tumor cell proliferation and play an important role in the occurrence of the BC.

Additionally, genetic alteration has been demonstrated to uncover gene expression, playing a vital role in the progression of cancer [31]. In this study, SRD5A3, DOLK, SRD5A1, and HSD17B3 appeared genetic alteration in BC, and alteration percentage varied from 2.7 to 8%. Alterations included missense mutation, splice mutation, truncating mutation, amplification and deep deletion. For the details, structural mutations of gene at specific genomic locations may alter its function and DNA copy number, which was essential in tumorigenesis [32]. As for SRD5A3 mutation analysis, it occurred nonsense mutation in Q96* protein domain. The potential effect of SRD5A3 genetic alteration on its mRNA expression needs further investigations.

In summary, we compared the SRD5A3 expression levels in BC group and normal group, and performed functional enrichment analysis, which may be useful to understand the underlying molecular mechanism of the BC occurrence. In addition, our results may be valuable to demonstrate a new biomarker for BC and may help develop novel early interventions in cancer treatment. Nevertheless, further investigations are required to validate the role of SRD5A3 expression in BC since all the data in our study were obtained from bioinformatics and lack of experiments.

## Conclusion

In conclusion, over-expression of SRD5A3 was revealed in BC tissues and high SRD5A3 expression was associated with poorer prognosis. Besides, SRD5A3 expression had a significant relationship with N stage and age. Further, SRD5A3 was involved in metabolism-associated pathway. And SRD5A3 serves as an oncogene and might function as a potential biomarker for prognosis and a therapeutic target for BC in the future.

## Data Availability

The datasets used and/or analyzed during the current study are available from the corresponding author on reasonable request.

## Abbreviations

BC: Breast cancer
SRD5A3: Steroid 5 alpha-reductase 3
TNBC: Triple-negative breast cancer
HER2+: Human epidermal growth factor receptor 2-positive
ER+: Estrogen receptor-positive
HCC: Human hepatocellular cancer
HPA: Human protein atlas
GO: Gene Oncology
KEGG: Kyoto Encyclopedia of Genes and Genomes
PPI: Protein-protein interaction
